# Editorial

**Published:** 1873-02

**Authors:** 


### EDITORIAL.

### Gutta Percha.

The practicability of substituting vulcanized gutta percha for hard rubber for dental plates is no longer a matter of uncertainty: it is an established fact. About one year ago Dr. E. Hale jr., of St. Louis, made a series of experiments with gutta percha, with the view of determining its availability as a substitute for rubber for artificial dentures. Such was his success, that in July last he became fully convinced that it was entirely feasible, and since that time he has been using it largely in practice: samples of dentures, the plates composed of vulcanized gutta percha, made by Dr. Hale, was exbibited at the American Dental Association, at Niagara Falls,. at its last meeting. We have just received a communication in which Dr. Hale says, "It is a complete success, and the profession in this city (St. Louis) and vicinity, are using it to the exclusion of rubber." To Dr. Hale is certainly due the regard and appreciation of the profession for his efforts in this direction.

During the last two months quite a number in the profession have been experimenting with this material; and many of them report with very satisfactory results.

Two objects are sought by these experiments, viz: to obtain a cheap base for artificial teeth that is better than rubber, and to thwart the Dental Vulcanite Company; both very commendable, and worth working for.

It was demonstrated twelve years ago that gutta percha could be vulcanized as well as rubber, but the Goodyear patent embraced it as well as rubber, and it was not then regarded as better than rubber, and being under the same embarrassment its use was discontinued, and it was not introduced to any considerable extent.

Gutta percha is not included in the Cumming's patent, or at least it was not a short time ago; it probably will be ere long!!

### Southworth's Operating Stool.

We have in use one of Southworth's operating stools, and take pleasure in recommending it as the best thing of the kind offered to the profession. It is very convenient; it has a range of elevation of the seat of eleven inches; there are two foot rests each separate from the other with a range of elevation of seventeen inches; these changes make it suitable for the use of any one. This is a fixture that every operator ought to have with his chair. Most operators stand while operating, but very many of them do so because they have no convenient arrangement for sitting. All operators would find it convenient and a great relief to sit occasionally whilst operating; and we doubt not many would sit much more with such an appliance as this. And in its more general use there would without doubt be a mitigation of some of the affections to which dentists are so much subject, with which many in the profession are but too well acquainted without a mention of them here.

We would advise every dentist to procure some arrangement by which he may sit occasionally, at least, while operating, and there is nothing as yet before the profession that will at all compare with Dr. Southworth's stool.

### Ohio Dental College—Spring Course.

The second spring term in this Institution will be opened on Tuesday, April 1st, and continue till June 1st.

There will be six lectures each week, one each day including Saturday. The remainder of the time will be devoted to clinical instruction and demonstration.

The course will be conducted with the view of giving the greatest amount of practical instruction in the shortest time; and will be especially adapted to the practitioner, who has been long enough engaged in the profession to know his weak points, and to be conscious of the fact that some assistance would do him good.

This course will be in charge of a corps of competent instructors.

The same text books are used as in the regular course.

All who have instruments for operating or tools for the mechanical departments should bring them.

Tickets for the course, $30.00.

There is no degree conferred from this course, neither does it answer the requirements of a course for graduation. We shall be pleased to receive an early notification from all who propose to attend.

J. TAFT, Dean.

### Mississippi Valley Dental Association.

The twenty-ninth annual meeting of the Mississippi Valley Dental Association will be held in the Ohio Dental College, beginning on Wednesday, March 5th, prox., at 10 o'clock A. M., and continuing three days.

### ORDER OF BUSINESS.

1st. Calling the Roll.

2d. Reading the Minutes.

3d. Reports of Standing Committees—Executive Committee—Committe on Membership—Committee on Ethics.

4th. Reports of Special Committees.

5th. Election of Members and Payment of Dues.

### ESSAYS AND DISCUSSIONS.

SUBJECTS—1st. Dental Legislation: Is it practicable? What benefits to the public and the profession may be expected by enforcing such laws?

2d. Artificial Dentistry: Should it be practiced as a specialty? What may be done to raise the standard of operations in this department?

3d. Filling Teeth: What may the patient reasonably expect shall be accomplished by the operation?

4th. Methods and materials most suitable to obtain the best results in filling teeth.

5th. Dr. Arthur's method of treatment and prevention of decay of the teeth.

6th. Conservative treatment of exposed or diseased dental pulps.

7th. Diseased conditions of the soft tissues of the mouth—their pathology and treatment.

8th. Dental Fees. What rules should govern the dentist in estimating the value of his professional services?

9th. The Dental Office. Ventilation—Light—The Use of Magnifying Lens.

10th. The use of mechanical power in operative dentistry.

Members are earnestly requested to prepare essays and papers to be read at this meeting. Those relating to the question under discussion will be read upon the introduction of the subject."

The election of officers will take place at 11 o'clock on the third day.

A large and interesting meeting is confidently expected, and a general invention is extended to all to be present.

H. A. SMITH,<br>

I. KNAP, *Executive Committee.*<br>

W. P. HORTON,

FRANK A. HUNTER, *Secretary.*

### Board of Examiners.

There will be a meeting of the State Board of Dental Examiners in the lecture room of the Ohio Dental College, Cincinnati, on Tuesday, the 4th of March prox., at 10 o'clock, A. M. It is desirable that all who wish to meet the Board will be promptly on hand at that time.

### College Association.

The annual meeting of the Ohio Dental College Association will be held in the College on Tuesday, March 4th, prox., at 2 o'clock P. M.

It is very desirable that there be a full attendance of the members. Annually the institution ought to receive the special attention of every member of the Association; then let no one be absent who can possibly be present.

### A Trial of Fifty Years.

The New York *Observer* has paseed through the ordeal, and starts out anew on the second fifty years with a larger list of readers and more numerous friends than ever. Such a steady course of prosperity is unexampled, and inspires confidence. We heartily rejoice in the great success of a paper which has always advocated those sound principles that underlie the foundations of society and good government. Orthodox in the truest sense, both in Church and State, its influence is always good. We see its publishers propose to give to every subscriber for 1873 an appropriately embellished *Jubilee Year-Book.* Those who subscribe will have no cause to regret the step. $3 a year. Sidney E. Morse & Co., 37 Park Row, New York.

### Obituary.

DIED on the morning of January 2d, at 4 o'clock A. M., Dr. L. A. Hendricks, of Milford, Ohio, in his 47th year, after a severe and lingering illness.

Dr. Hendricks was educated for the medical profession, and engaged in its practice for several years, and with very marked success. About fifteen years ago he took a thorough course preparatory to practice of dentistry, since which he has been engaged in dental practice, except whilst he was in the army, which was most of the time during the war. During this time he endured great hardship; and doubtless there contracted the germs of that disease by which he was finally cut down. He has for most of the time since he left the army been an invalid.

Dr. Hendricks was a kind-hearted, genial, unassuming, Christian gentleman, who by these virtues and his talents commanded and received the respect and love of all who knew him.

In his demise, the profession has lost a noble member, society a good man, and his family a loving husband, and a kind and indulgent father.

May such virtues as he possessed be largely imitated.

### Obituary.

Died on the 4th inst., at 6 o'clock A. M., Dr. Henry Schmidt Schaller, in his thirty-seventh year, at his late residence in this city. He was born at Constanz, Germany, and came to this country in 1855.

He was a graduate of the Philadelphia Dental College, and has practiced his profession in this city during the last ten years.

He was held in high esteem by all who knew him.

The following action was had by the profession of this city upon learning of his death.

At a meeting of the dentists of this city, held at the office of Dr. A. Berry, on the 5th instant, the following resolutions were adopted:

"WHEREAS, We have heard of the very sudden and unexpected death of Dr. H. Schmit Schaller, of this city, with deep regret and sorrow; and

"Whereas, By this visitation of Divine Providence, we are deprived of the society of our late brother and associate, we consider it proper and right to give such expression of our feelings as are appropriate to the occasion.

"Resolved, That in the death of Dr. Schaller the profession has lost a worthy and honorable member, and his loss will be deeply felt by all who knew him.

"Resolved, That we tender the stricken family and friends our heartfelt condolence and sympathy, and express the sadness we can not but share with them.

"Resolved, That a copy of these resolutions be sent to the family of our deceased brother, and that the profession in this city attend the funeral in a body.

"H. R. SMITH, Chairman.

"J. L. TAYLOR, Secretary.

